# Supplementary material for: Neutrophil Macrophage Crosstalk via Extracellular Vesicles Drives Reverse Migration in a Fully Human Model of Wound Healing
Source: Adv Sci (Weinh). 2025 May 31;12(31):e01036. doi: 10.1002/advs.202501036 (PMC12376556; doi:10.1002/advs.202501036)
Supplement: Supplementary file 1 — Supporting Information [file ADVS-12-e01036-s009.docx]

**SUPPLEMENTARY FIGURES**

**Neutrophil macrophage crosstalk via extracellular vesicles drives reverse migration in a**

**fully human model of wound healing.**

**Kehinde Adebayo Babatunde^1^, Babatunde Fatimat Oluwadamilola^2^, Adeel Ahmed^1^, Wilmara Salgado-Pabon^2^, David J Beebe^1,3,4^ and Sheena C Kerr^4*^.**

**^1^Department of Pathology & Laboratory Medicine, University of Wisconsin, Madison, WI, USA.**

**^2^Department of Veterinary Medicine, University of Wisconsin, Madison, WI, USA.**

**^3^Department of Biomedical Engineering, University of Wisconsin-Madison, Madison, WI 53715, USA.**

**^4^Carbone Cancer Center, University of Wisconsin, Madison, WI, USA.**

***Corresponding author: Sheena Kerr (skerr2@wisc.edu)**

**
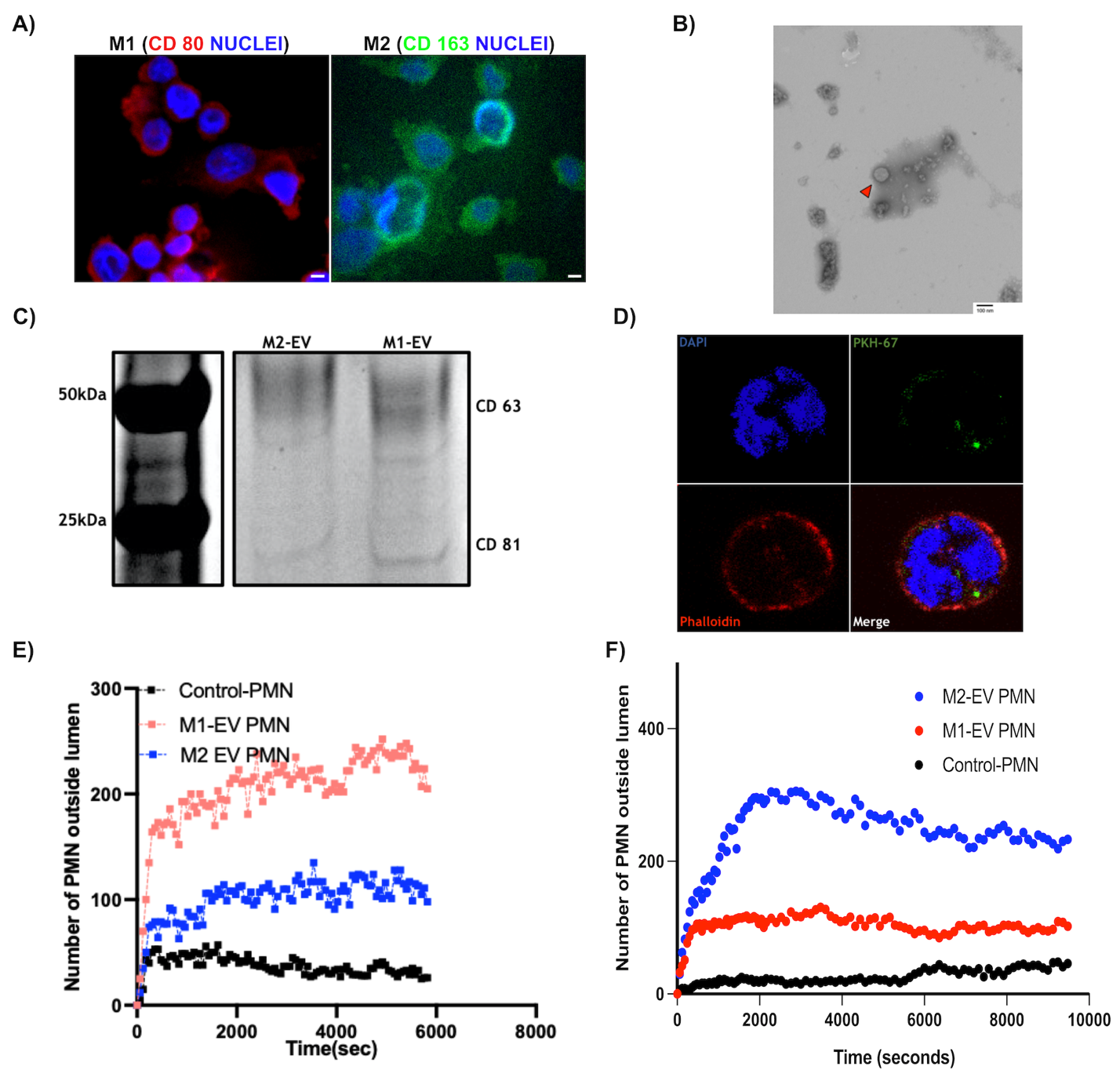
**

**FIGURE 1:** **Macrophage polarization and EV characterization** A) Representative images of monocyte differentiated macrophage polarization to M1 macrophage (showing the expression of CD 80 marker) and M2 macrophage (showing the expression of CD 163 marker) *in vitro*. Scale bar is 20µm. B) Electron microscopy image of M-EVs. Mag: 66000x. Scale bar: 50nm. Red arrow points to the M-EV. C) WB of M-EV markers showing the protein ladder. D) Immunofluorescence images showing neutrophil with M-EVs; Neutrophil nuclei (DAPI-Blue), Actin filament (Phalloidin-Red) and M-EVs (PKH-67-Green). Scale bar: 10µm. Migratory dynamics and number of neutrophils outside the lumen during E) nSI and F) SI.

**A)**


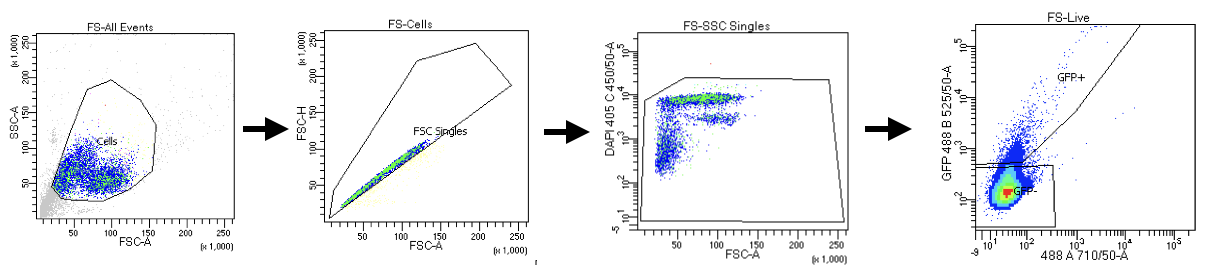

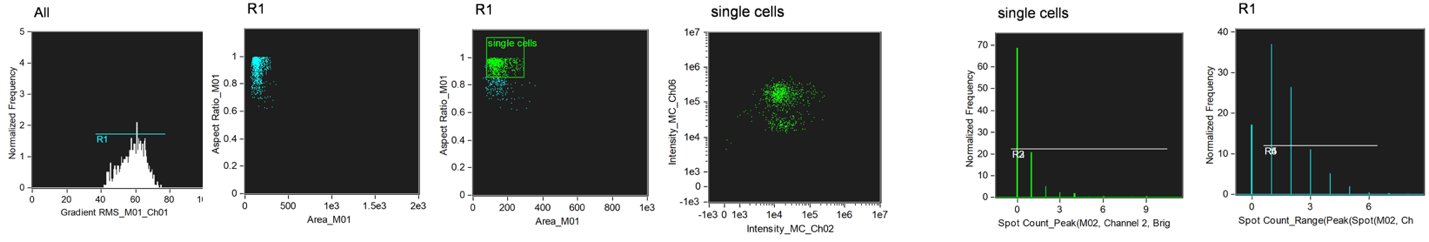


**B)**

**FIGURE 2:** **Image stream analysis of neutrophil-EV interactions**. A) Image stream cytometry gating analysis showing population of neutrophil with M-EVs and without M-EVs. B) Image stream software analysis of neutrophils showing different counts of M-EVs being uptaken by neutrophils.

**
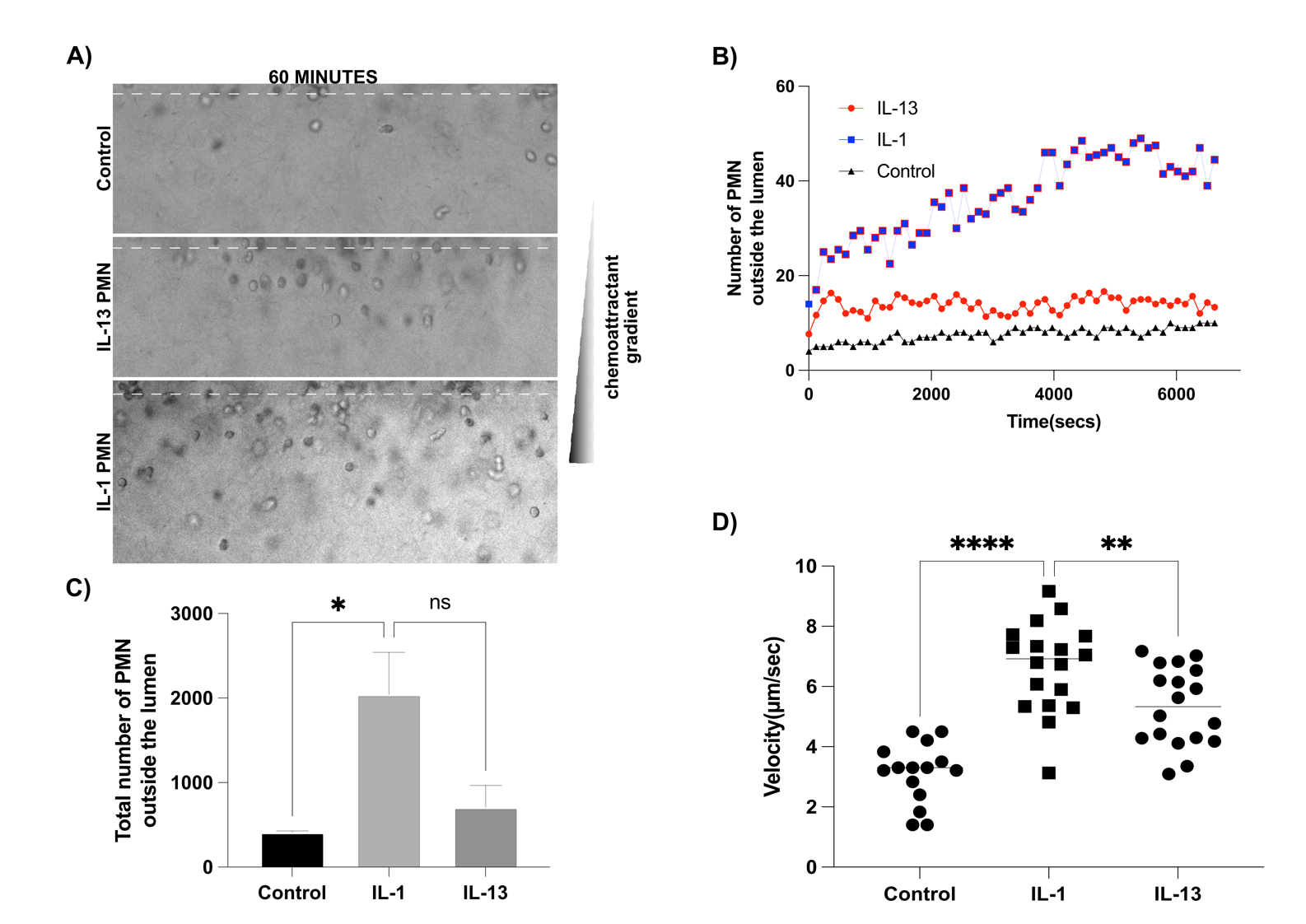
FIGURE 3: Chemotaxis of pro- and anti-inflammatory chemokine stimulated neutrophils**. A) Brightfield images of neutrophils migrating out of lumen (white dashed line). B) Quantification of the number of neutrophils migrating after IL-1 or IL-13 stimulation compared to unstimulated control over time. C) Total number of migrated neutrophils after 2 hours of migration. D) Velocity of migrating neutrophils.


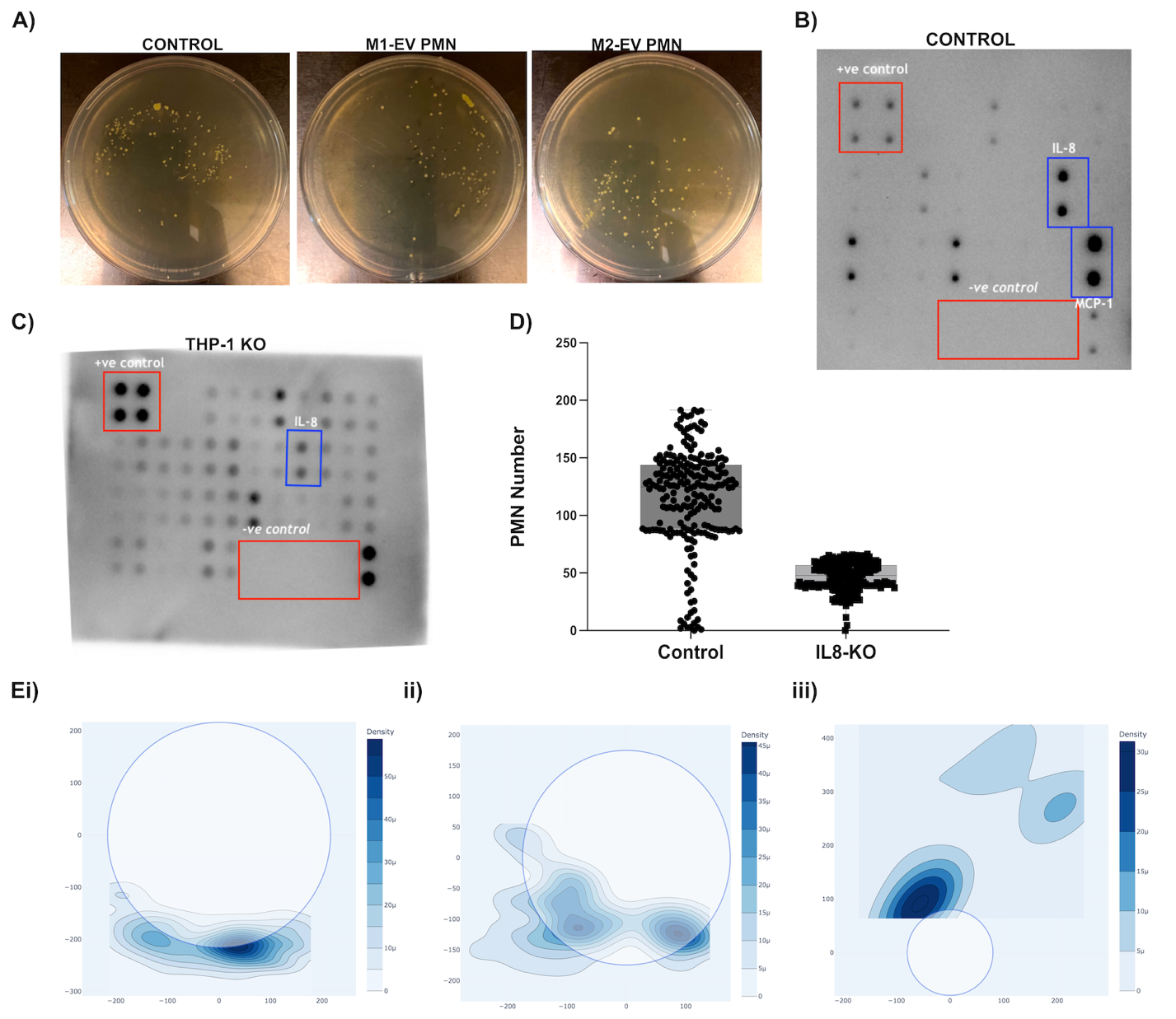


**FIGURE 4:** A) Representative image of viable bacteria agar plate after incubation with control, M1-EV PMN and M2-EV PMN. Chemokine array analysis image showing IL-8 cargo in B) Control THP-1 cells and C) IL-8 THP-1 KO cells. D) Graph showing number of neutrophils that responded to THP-1 like macrophage cells and IL-8 KO THP-1 like macrophage cells (chemotaxis assay). E) Representative heatmap of the migratory tracks in i) M1-EVs treated neutrophils, ii) M2-EV treated neutrophils and iii) IL8 KO EV treated neutrophils toward the SI site. Black circles represent the boundary of the SI site.
